# Genetic Alterations in a Large Population of Italian Patients Affected by Neurodevelopmental Disorders

**DOI:** 10.3390/genes15040427

**Published:** 2024-03-28

**Authors:** Annaluisa Ranieri, Ilaria La Monica, Maria Rosaria Di Iorio, Barbara Lombardo, Lucio Pastore

**Affiliations:** 1 CEINGE-Biotecnologie Avanzate Franco Salvatore, via G. Salvatore 486, 80145 Naples, Italy; ranieria@ceinge.unina.it (A.R.); lamonica@ceinge.unina.it (I.L.M.); diiorio@ceinge.unina.it (M.R.D.I.); lucio.pastore@unina.it (L.P.); 2 Dipartimento di Medicina Molecolare e Biotecnologie Mediche, Università di Napoli Federico II, via Sergio Pansini 5, 80131 Naples, Italy

**Keywords:** array-CGH, autism spectrum disorders, neurodevelopmental disorders, copy number variants

## Abstract

Neurodevelopmental disorders are a group of complex multifactorial disorders characterized by cognitive impairment, communication deficits, abnormal behaviour, and/or motor skills resulting from abnormal neural development. Copy number variants (CNVs) are genetic alterations often associated with neurodevelopmental disorders. We evaluated the diagnostic efficacy of the array-comparative genomic hybridization (a-CGH) method and its relevance as a routine diagnostic test in patients with neurodevelopmental disorders for the identification of the molecular alterations underlying or contributing to the clinical manifestations. In the present study, we analysed 1800 subjects with neurodevelopmental disorders using a CGH microarray. We identified 208 (7%) pathogenetic CNVs, 2202 (78%) variants of uncertain significance (VOUS), and 504 (18%) benign CNVs in the 1800 patients analysed. Some alterations contain genes potentially related to neurodevelopmental disorders including *CHRNA7*, *ANKS1B*, *ANKRD11*, *RBFOX1*, *ASTN2*, *GABRG3*, *SHANK2*, *KIF1A SETBP1*, *SNTG2*, *CTNNA2*, *TOP3B*, *CNTN4*, *CNTN5*, and *CNTN6*. The identification of interesting significant genes related to neurological disorders with a-CGH is therefore an essential step in the diagnostic procedure, allowing a better understanding of both the pathophysiology of these disorders and the mechanisms underlying their clinical manifestations.

## 1. Introduction

Molecular cytogenetic techniques, such as the array-comparative genomic hybridization (a-CGH) method, have increased our ability to identify clinically relevant submicroscopic copy number variants (CNVs) in patients with neurodevelopmental disorders (NDDs), such as autism spectrum disorder (ASD), intellectual disability (ID), attention deficit hyperactivity disorder (ADHD), psychomotor developmental delay, specific learning disorder, communication disorders, speech delay, epilepsy, and schizophrenia [1,2,3]. NDDs are a group of heterogeneous conditions whose onset and clinical expression occur during childhood or adolescence and are characterized by impairment in cognition, communication, adaptive behaviour, and psychomotor skills resulting from abnormal brain development [4,5]. Such disorders represent a serious health problem in our society, affecting 3% of children worldwide [6]. NDDs have a strong genetic basis; however, additional causal environmental factors have also been identified [7]. These factors may interact during brain development causing disorders that to date have not been fully elucidated. The influence of environmental insults and the presence of genetic alterations during any of these stages can alter brain development and cause these disorders [8,9]. Many studies suggest the presence of shared molecular pathways underlying multiple clinical signs typical of NDDs; in fact, the comorbidity of two or more of these disorders is often found in the same individual [10]. Identifying the pathogenetic mechanisms common to different NDDs will help explain the aforementioned comorbidity and will eventually lead to the identification of effective therapies [6,11]. CNVs represent the genetic aberrations most related to NDDs [12,13,14]. The identification of CNVs and mutations in specific genes as significant causative factors in NDDs, via a-CGH and exome sequencing or genome-wide next-generation sequencing (NGS) analyses, is critical for accurate genetic counselling and represents an essential first step toward a better understanding of the molecular pathways underlying these disorders [15,16,17,18,19]. In particular, a-CGH is currently considered a first-level diagnostic tool for identifying neurodevelopmental disorders with a significantly higher diagnostic performance (5–10%) than conventional karyotype analysis [12,20].

In the present study, we analysed subjects with ASD, ID, speech delay, psychomotor developmental delay, ADHD, epilepsy, schizophrenia, and other neurological disorders using a-CGH. We evaluated diagnostic efficacy of a-CGH and its importance as a routine genetic test in patients with NDDs to identify molecular alterations underlying or contributing to clinical manifestation [21]. Specifically, we have identified in our patients and, subsequently, studied through database and literature support the major pathogenic CNVs and variants of uncertain significance (VOUS) containing in some cases a single gene potentially related to their disorders. We also believe that the early diagnosis of NDDs, through careful observation of early signs that may occur during childhood and appropriate molecular diagnostics, can potentially allow significant changes in the clinical evolution of these disorders [22]. Early specific therapies can possibly improve their clinical progress and positively influence their evolution, preventing or reducing their evolution into more severe psychiatric disorders later in life [6].

## 2. Materials and Methods

### 2.1. Patients

In the period between January 2019 and March 2023, we evaluated by a-CGH a cohort of 1800 paediatric and adolescent patients aged between a few days and 18 years old; patients were affected by different neurodevelopmental disorders with a male-to-female ratio of 2.5:1. The patients were referred from different departments, in particular, 2% from the Dipartimento di Neuropsichiatria Infantile, Università della Campania “Luigi Vanvitelli” (A.O.U. Luigi Vanvitelli), Napoli, 39% from Dipartimento di Neuropsichiatria Infantile, Università degli Studi di Napoli “Federico II” (A.O.U. Federico II), Napoli, 25% from Dipartimento di Pediatria, Università degli Studi di Napoli “Federico II”, (A.O.U. Federico II), Napoli, 4% from Dipartimento di Genetica Medica, Azienda Ospedaliera di Rilievo Nazionale Santobono Pausilipon (A.O.R.N. Santobono Pausilipon), Napoli, 3% from Dipartimento di Genetica Medica, Azienda Ospedaliera di Rilievo Nazionale Antonio Cardarelli, (A.O.R.N. Antonio Cardarelli), Napoli, 11% from Dipartimento di Neuropsichiatria Infantile, Azienda Ospedaliera San Giovanni di Dio e Ruggi d’Aragona (A.O.U. San Giovanni di Dio e Ruggi d’Aragona), Salerno, and 16% from DAI Medicina di Laboratorio e Trasfusionale, Azienda Ospedaliera Federico II, Napoli (Ambulatorio DAIMELAB A.O.U. Federico II). When available, diagnostic investigations were conducted on the parents of the patients analysed, in order to determine the transmission or de novo origin of the identified CNVs.

Developmental delay and ID were evaluated by using different methods depending on the patients’ age. In early childhood, before the age of three, the assessment of motor and cognitive developmental was evaluated using the Bayley Scales of Infant Development and Toddler Development, the Griffith Mental Development Scales, and the Autism Diagnostic Observation Schedule, Second Edition (ADOS-2). After three years of age, the Autism Diagnostic Interview-Revised (ADI-R) was performed; for children six years old and older, Wechsler Intelligence Scale for Children (WISC) for ID, the Raven’s Matrices, and the Vineland Adaptive Behavior Scales for the adaptive quotient were used. All patients gave their written informed consent to the study, which was performed according to the Helsinki II Declaration and was approved by the Ethics Committee of our Faculty of Medicine (authorization no. 193/06, 25 October 2006; amendment no. 193/06/ESES1, 1 October 2014).

### 2.2. Genetic Testing

Patients’ DNA and their parents’ DNA, when available, was isolated from peripheral blood samples using the RSC Blood DNA Kit (Promega, Madison, WI, USA), using manufacturer’s protocol. DNA quality and concentration were determined with a NanoDrop 2000 spectrophotometer (ThermoFisher Scientific Inc., Bartlesville, OK, USA). Following extraction, parents’ DNA and their parents’ DNA were processed according to the Agilent protocol (Version 7.5, June 2016) using Human Reference DNA (Promega, Madison, WI, USA). In particular, chromosomal microarray analysis (CMA) was performed using SurePrint G3 Human CGH Array 4 × 180 K (Agilent Technologies, Santa Clara, CA, USA), according to the manufacturers’ recommendation. The Agilent SurePrint G3 Human CGH Array 4 × 180 K has an average spacing of 13 kb, allowing an average resolution of 25 kb. The array images were acquired using the Agilent laser scanner G2600D. Image files were quantified, and data were visualized by using Agilent’s Cytogenomics software version 4.0.3.12 (Agilent Technologies, Santa Clara, CA, USA). Finally, the detected pathogenic and likely pathogenic CNVs were validated using quantitative real-time polymerase chain reaction (RT-qPCR) technique. RT-qPCR was performed using the SYBR Green method and implemented in the Applied Biosystems 7900HT Fast Real-time PCR system, following the manufacturer’s protocol (Applied Biosystems, Life of technologies). Primers for each pathogenic/probably pathogenic CNV were designed using Primer Express 2.0 software (Applied Biosystems, Carlsbad, CA, USA); primers selected from the ZNF80 gene were used to generate a normalizing amplicon. Assays were performed in a total volume of 10 μL in duplicate in 96-well optical reaction plates (Applied Biosystems). For each sample, a relative quantification was carried out using 9 genomic DNA samples (ctrls). A comparative 2^−∆∆Ct^ method was used to validate the rearrangements identified by a-CGH in this study [23].

### 2.3. Data Analysis

The analysis algorithm for CNVs was set in Agilent’s Cytogenomics version 4.0.3.12 ADM2 analysis software with a threshold of 6, with the minimum number of probes considered for the inclusion of a CNV equal to 3, and the Minimum Absolute Average Log Ratio of Region equal to 0.25. All CNVs of the same size as those detected with a minimum of 3 probes were analysed. The discovered CNVs were analysed and compared with aberrations present in a number of databases including: Database of Genomic Variants (DGV; http://dgv.tcag.ca/dgv/app/home, 15 February 2024), Database of Chromosomal Imbalance and Phenotype in Humans (DECIPHER; https://decipher.sanger.ac.uk/, 15 February 2024), ClinVar (National Center for Biotechnology Information, U.S. National Library of Medicine; http://www.ncbi.nlm.nih.gov/clinvar/, 15 February 2024), ClinGen (https://clinicalgenome.org, 15 February 2024), The Human Gene Database, GeneCards (https://www.genecards.org/, 15 February 2024), and Online Mendelian Inheritance in Man (OMIM; https://www.omim.org/, 15 February 2024). CNVs were classified into three groups (pathogenic, VOUS, and benign) according to the American College of Medical Genetics and Genomics (ACMG) standards and guidelines [24]. Benign CNVs have typically been reported in multiple peer-reviewed publications or annotated in curated databases as benign and are usually present in >1% of the population. Pathogenic CNVs included alterations with consistent clinical phenotypes across multiple peer-reviewed publications, well-documented penetrance, and expressivity and with almost one gene known to be dosage sensitive. VOUS represents a broad category and may include findings that are later demonstrated with additional evidence to be either pathogenic or benign. Moreover, we analysed our results by using the Simons Foundation Autism Research Initiative database gene autism (SFARI), in which 2290 different CNV regions and 1416 genes are reported and associated with ASD in different types of studies (update October 2023; https://gene.sfari.org/). The analysis was carried out by evaluating the correlation of the alterations with the main diagnostic suspicion present on the parents’ request, in order to avoid confusing interpretations of the results.

## 3. Results

**Figure 1:**
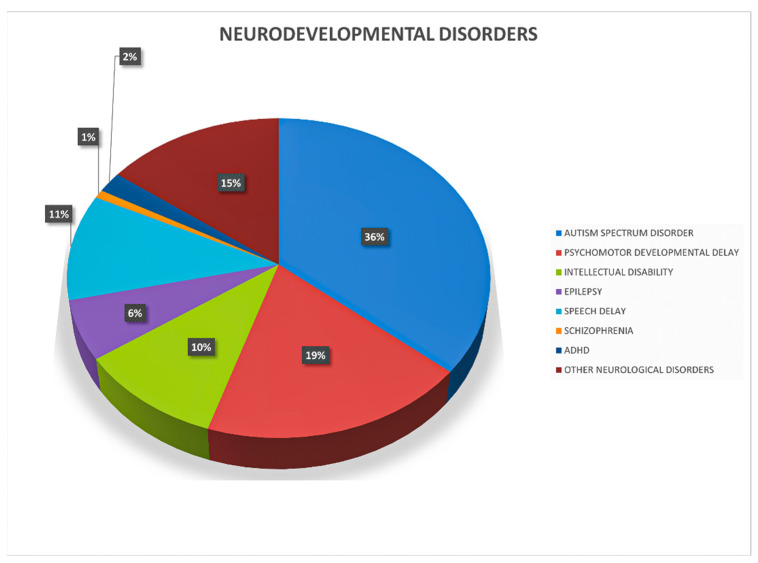
Frequency of main diagnostic suspects reported on the request of our patients.

**Figure 2:**
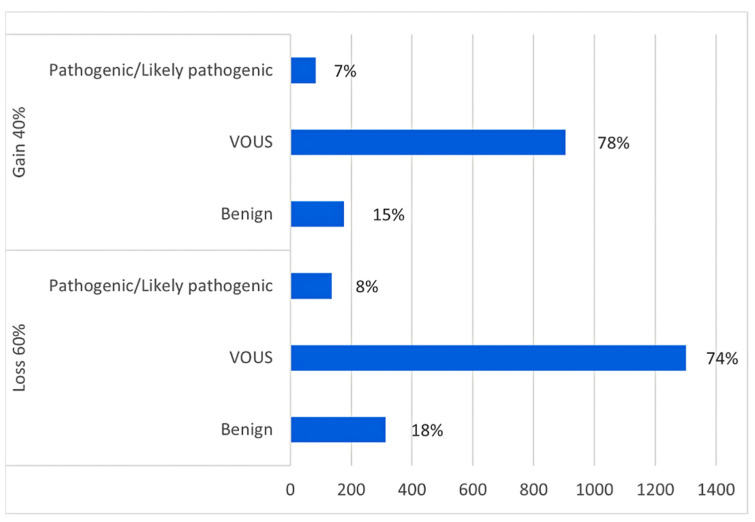
Comparison of the number and significance of loss and gain identified in patients with neurodevelopmental disorders.

Among the 1800 patients who came to our attention, 649 (36%) had ASD, 341 (19%) psychomotor developmental delay, 187 (10%) ID, 194 (11%) speech delay, 38 (2%) ADHD, 15 (1%) schizophrenia, 109 (6%) epilepsy, and 267 (15%) other neurological disorders (Figure 1). Since it was not possible to carry out a complete clinical evaluation and phenotyping on the patients, they were divided and classified on the basis of the main diagnostic suspicion reported on request. For this reason, it must be considered that comorbidities are common in neurodevelopmental disorders and the final diagnosis may include multiple phenotypic traits. We identified 208 (7%) pathogenic CNVs, 2202 (76%) VOUS, and 504 (17%) benign CNVs in our patients (all informations are available in the Supplementary Materials). In total, the number of deletions (1750, 60%) identified in patients is slightly higher than the number of duplications (1164, 40%). Microdeletions were more frequently classified as pathogenic or likely pathogenic compared to microduplications (60% vs. 40%, Figure 2); however, their sizes did not differ significantly. Among the 194 patients with 208 pathogenic/likely pathogenic CNVs, 33% of CNVs (68/208) were of de novo origin, 14% of CNVs (29/208) were inherited from a parent. In the remaining cases, parental DNA was not available for analysis.

In addition, 76% of all a-CGH findings (2202/2914) were classified as VOUS. Among the VOUS for which it was possible perform analysis on parental DNA, 21.9% of CNVs (482/2202) were of de novo origin, and 28% of CNVs (626/2202) were inherited. CNVs in 26.4% of the cases analysed were classified as likely benign with 504 CNVs in 476 patients (476/1800). In general, the likely benign CNVs found are poor in genes and they are most likely not correlated with the phenotype of the patients. A majority of benign CNVs were found to be inherited from unaffected parents when they were tested: in fact, 58% of the likely benign CNVs (292/504) were inherited, while 36 CNVs were of de novo origin.

Among the pathogenic and likely pathogenic alterations observed in our patients, we identified microdeletion and microduplication associated with specific syndromes: microdeletion 1q21.1-q21.2 (1q21.1 Deletion syndrome OMIM#612474, Thrombocytopenia-absent radius syndrome OMIM#274000), microduplication 1q21.1-q21.2 (1q21.1 Duplication syndrome OMIM# 612475), microduplication 15q11.2-q13.1 (Duplication 15q11-q13 syndrome OMIM#608636, Angelman syndrome OMIM#105830, Prader–Willi syndrome OMIM#176270), microdeletion 16p11.2 (16p11.2 deletion syndrome, OMIM#611913), microduplication 16p11.2 (16p11.2 duplication syndrome OMIM#614671), microdeletion 22q11.21 (DiGeorge syndrome OMIM#188400, Velocardiofacial syndrome OMIM#192430), microduplication 22q11.21 (22q11.2 microduplication syndrome OMIM#608363, DiGeorge syndrome OMIM#188400, Velocardiofacial syndrome OMIM#192430), microdeletion Xp21.1 (Duchenne muscular dystrophy OMIM#310200, Becker muscular dystrophy OMIM#300376), microduplication 17p12 (Charcot–Marie-Tooth disease, type 1A OMIM#118220), and microdeletion 17p11.2 (Smith–Magenis syndrome OMIM #182290) (Table 1). Instead, among the pathogenic/likely pathogenic CNVs, being unique non-recurrent and not associated with syndromes, we have identified interesting CNVs such as microduplication 11q13.1-q13.2, microduplication 5q14.3, microdeletion Xq28, microdeletion 13q21.32-q31.1, microdeletion 2p16.3, microduplication 20p12.3-p12.1, and microdeletion 15q26.3 (Table 2).

Among the VOUS observed in patients (Table 3), we selected by detailed gene content analysis CNVs of uncertain clinical significance structural containing genes significantly related to neurodevelopmental disorders, such as Ankyrin Repeat And Sterile Alpha Motif Domain-Containing 1B (*ANKS1B*, OMIM*607815), located in 12q23.1; Ankyrin Repeat Domain 11 (*ANKRD11*, OMIM*611192), located in 16q24.3; RNA Binding Fox-1 Homolog 1 (*RBFOX1*, OMIM*605104), located in 16p13.3; Cholinergic Receptor Nicotinic Alpha 7 Subunit (*CHRNA7*, OMIM*118511), located in 15q13.3; Gamma-Aminobutyric Acid Type A Receptor Subunit Gamma3 (*GABRG3*, OMIM*600233), located in 15q12; Astrotactin 2 (*ASTN2*, OMIM*612856), located in 9q33.1; SH3 And Multiple Ankyrin Repeat Domains 2 (*SHANK2*, OMIM*603290), located in 11q13.3-q13.4; Kinesin Family Member 1A (*KIF1A*, OMIM*601255) located in 2q37.3; Set-binding Protein 1 (*SETBP1*, OMIM*611060), located in 18q12.3; Syntrophin-gamma-2 (*SNTG2*, OMIM*608715), located in 2p25.3; Catenin, alpha-2 (*CTNNA2*, OMIM*114025), located in 2p12; Topoisomerase, DNA, III, beta (*TOP3B*, OMIM*603582), located in 22q11.22; Contactin 4 (*CNTN4*, OMIM*607280), located in 3p26.3-p26.2; Contactin 6 (*CNTN6*, OMIM*607220), located in 3p26.3; and Contactin 5 (*CNTN5*, OMIM*607219), located in 11q22.1.

## 4. Discussion

A-CGH is an extremely relevant diagnostic step for the discovery of new submicroscopic rearrangements involved in the pathogenesis of NDDs; its diagnostic yield has rapidly increased so that it was adopted and recommended as a first-level clinical diagnostic test for such disorders [25,26]. In order to adequately determine the role of the CNVs detected in patients, we considered their type (deletion or duplication), size, gene content, inheritance, and information present in public databases and in the literature. The identification of new microdeletions and microduplications in the human genome represents an important challenge to determine the role and clinical impact of these chromosomal rearrangements [27]. For this reason, during our 4-year study, we analysed patients with NDDs using a-CGH, identifying CNVs containing interesting genes, potentially related to the phenotypic characteristics found in our patients and which could contribute to clarifying the pathogenesis of these disorders. Many pathogenic CNVs result in similar clinical manifestations such as ID and ASD, but may differ in the occurrence of specific and different phenotypic traits for each subject. In fact, the impact of different variants in allelic loci present in each individual genome may act as modifiers; in addition, different expression of the alleles could also explain this phenomenon [28]. In particular, these phenomena could clarify cases in which patients inherit pathogenetic mutations from healthy parents causing incomplete penetrance or variable expressivity of the genes included in the detected CNVs [28]. For example, in patients with CNVs classified as pathogenic/likely pathogenic, we identified two cases of 1q21.1-q21.2 microdeletion of maternal origin and one case of paternal origin, one case of 1q21.1-q21.2 microduplication of paternal origin, one case of 22q11.21 microdeletion inherited from the father, one case of 22q11.21 microduplication inherited from the mother, one case of 16p11.2 microdeletion of maternal origin, one case of 17p12 microduplication of paternal origin, one case of 17p11.2 microdeletion inherited from the father, one case of microdeletion 15q26.3 of paternal origin, one case of 2p16.3 microdeletion of maternal origin, and one case of paternal origin. In these cases, the modifying impact of multiple loci in the human genome could contribute to explain the more severe phenotype found in children compared to their parents or the variability of phenotypes among children who share the same CNVs [29]. Paternal or maternal inheritance of a CNV should also consider a possible source of variability due to the possible presence of imprinted genes.

Among the genes contained in the VOUS, we focused our attention on a small number of genes, not well characterized on databases and in the literature, but which were present in the SFARI databases and correlated with the development of phenotypic features present in NDDs; this approach can lead to the identification of new causative or potentially causative genes that contribute to elucidate the etiopathogenesis of these disorders.

The first interesting case that we have selected is an 8-year-old male child with ASD, a communicative-relational disorder in the presence of nonverbal intellectual functioning in the normal range, slow language evolution, discontinuous and fleeting gaze attachment, and epilepsy with continuous spike waves in sleep. We identified a deletion in the 12q23.1 region of 180.0 kb extent, partially containing the Ankyrin Repeat and Sterile Alpha Motif Domain-Containing 1B (*ANKS1B*) gene. This deletion involves intron 9, exon 9, and intron 8 of the *ANKS1B* gene; this alteration appeared to be de novo after an in-depth diagnostic investigation conducted on the parents. In addition, smaller deletions involving only intronic regions were identified in three patients with ASD and one patient with ID, ADHD, and dysmorphic features. Only in one of the four cases could the maternal origin of the deletion containing *ANKS1B* be observed. The *ANKS1B* gene encodes for the protein AIDA-1, highly expressed in the brain, and enriched in hippocampal and cerebellar regions; AIDA-1 is one of the most abundant proteins at neuronal synapses [30], located as a central postsynaptic density protein and interacting with PSD95 in a complex that contains other factors associated with NDDs, including *GRIN2B*, *SYNGAP1*, and *NLGN* [31]. Mouse models with heterozygous deletions in the *ANKS1B* gene have shown a number of distinctive behavioural signs typical of autism [32,33]. Monogenic CNVs including the *ANKS1B* gene have been identified in individuals showing a spectrum of neurodevelopmental phenotypes, including ASD, ADHD, and motor and language deficits [30,34,35]. The *ANKS1B* gene is present in the SFARI database with a 2.1S, Strong Candidate, Syndromic score with evidence that mutations in this gene have an important functional effect and are correlated with an increased risk of developing ASD. Therefore, *ANKS1B* could represent a crucial gene in which rare variants with loss of function cause NDDs, including ASD, ADHD, and deficits in speech and motor function.

Another CNV that we have evaluated consists of an approximately 43 kb duplication on chromosome 18 in the q12.3 region encompassing the SET binding protein 1 (*SETBP1*) gene, identified in a 4-year-old male patient with a NDD characterized by growth and psychomotor developmental delay, behavioural disturbances, and language regression from 18 months of age. The duplication partially includes intron 5 and exon 6 of the gene *SETBP1*; analysis of parental DNA was not possible, so we could not establish whether it was a de novo alteration. *SETBP1* has an important role in controlling forebrain progenitor expansion and neurogenic differentiation [36]. The *SETBP1* gene is associated with several NDDs; in fact, haploinsufficiency due to deletions in the heterozygosity of the gene or mutations with the production of a nonfunctioning protein cause a dysfunction-related disorder of the *SETBP1* gene, characterized by hypotonia and mild motor developmental delay; intellectual abilities ranging from normal to severely impaired; speech and language disorders; behavioural problems; and mild dysmorphic [37,38,39]. Its strong association with developmental delay along with language impairment makes *SETBP1* a new candidate gene for NDDs [40,41]. This gene is present in SFARI with a score of 1.1, high confidence, indicating a clear implication in the development of ASD. Specifically, duplications or mutations of the *SETBP1* gene likely result in a gain of function with subsequent degradation or production of a dominant negative suggesting a possible role of *SETBP1* gene duplication in the development of the phenotype found in the patient [36].

In addition, we identified and studied an alteration in the Astrotactin 2 (*ASTN2*) gene, found in a 6-year-old male patient diagnosed with ASD, characterized by significant difficulties in social interaction and communication with a tendency toward isolation, and also the presence of severe psychomotor instability and some motor stereotypies. The CNV present in our patient is a de novo deletion of extent 167.9 kb present in the 9q33.1 region involving intron 1, exon 2, and partially intron 2 of the *ASTN2* gene. *ASTN2* encodes for an integral membrane protein, expressed in the brain, that plays a central role in neural-glial adhesion during neuronal migration and synaptic strength through the trafficking and degradation of surface proteins [42]. *ASTN2* deletions has been identified in patients with ASD, ADHD, and schizophrenia, highlighting how *ASTN2* alterations are a risk factors for NDDs [43,44]. *ASTN2* knockout mice showed an increase in exploratory activity in a new environment, social behaviour and impulsivity, or a release of anxious and depressed behaviours [45]. Such behaviour is also seen in patients with ADHD and ASD, who are often unable to adapt to new situations [46,47]. According to the SFARI databases, *ASTN2* is reported as a strong candidate with a score of 2.1, which demonstrates that the variants present in this gene could have a functional effect and correlate with an increased risk of developing disorders such as ASD, especially due to the role of *ASTN2* in the modulation of synaptic activity during neural development.

Genetic variants involving a gamma-aminobutyric acid (GABA) receptor have been identified in patients with NDDs. In fact, we found a deletion in the 15q12 region encompassing the Gamma-Aminobutyric Acid Type A Receptor Subunit Gamma3 (*GABRG3*) gene in three ASD patients. GABA is the major inhibitory neurotransmitter in the mammalian brain where it acts on GABA-A receptors, which are ligand-gated chloride channels. Deletions or duplications of the 15q11-13 region containing the *GABR* genes are related to multiple neurological disorders including Prader–Willi syndrome (PWS), Angelman syndrome (AS), and autism [48]. In particular, we found in a 4 years old male patient, with ASD characterized by relational difficulties, absent eye contact, and severely impaired communication with indicative gesture and absent verbal language, a deletion of 47.0 kb involving intron 3 of the *GABRG3* gene. The unavailability of parents’ DNA prevented us from clarifying whether it was a de novo alteration. Studies in rat models have pointed out that the neurotransmitter GABA appears early in neurogenesis; during embryogenesis, GABAergic neurons become widespread in all regions of the developing brain and spinal cord [49,50,51]. A similar pattern emerges in the developing human CNS, with a transient and widespread distribution of GABAergic neurons [52]. This suggests that GABA plays a role in neurogenesis before becoming the main fast-acting inhibitory neurotransmitter in the brain. The subunits that make up GABAA receptors have also been shown to change during development [53]. In a pilot study, the role of some genetic variants of the GABA type A receptor subunits *GABRB3*, *GABRG3*, and *GABRA5* in the aetiology of ASD was investigated [54], supporting the hypothesis that ASD may be also caused by GABAergic dysfunctions [55]. In fact, *GABRG3* gene is reported in SFARI as a strong candidate with a score of 2.1 that correlates with an increased risk of developing NDDs such as ASD.

From the analysis carried out in a 15-year-old female autistic patient, with impaired pragmatic communication, difficulty interacting with her peers due to a severe deficit in social skills, and a strong state of anxious activation with isolation and obsessive behaviours, we identified a paternal duplication in the 2q37.3 region of 52.7 kb that partially containing the Kinesin Family Member 1A (*KIF1A*) gene, which aroused our particular interest. This duplication of paternal origin involves from intron 27 to intron 45 and from exon 28 to exon 46 of the *KIF1A* gene. The *KIF1A* gene encodes for a microtubule-dependent motor protein responsible for fast anterograde transport of synaptic vesicle precursors in neurons [56]. KIF1A is a motor protein expressed exclusively in the brain; in fact, several studies have shown that de novo pathogenic variants determine an increased risk of ASD [57,58]. There is therefore a link between *KIF1A* mutations and ASD. Notably, de novo dominant mutations in the motor domain have been reported in association with complex phenotypes characterized by ID and the variable presence of cerebellar atrophy, spastic paraparesis, hyperactivity, and epilepsy [57,58,59]. *KIF1A* is listed in the SFARI database as a strong syndromic candidate with score 2.1S, associated with an increased risk of developing ASD.

Furthermore, among the alterations evaluated we observed a paternally derived duplication in the 2p25.3 region of size 457.1 kb in a 9-year-old patient with poor social interaction, severe communication delay, hyperresponsiveness to external stimuli, poor orientation, hyperactivity, and episodes of delirium consistent with a diagnosis of ASD made at age 4 years. This duplication included exons 1–6 of the Syntrophin-γ2 (*SNTG2*) gene. CNVs of different sizes containing the *SNTG2* gene were also found in four patients with ASD, two patients with ID, and one patient with psychomotor developmental delay and speech delay. It was only possible in three cases to perform an in-depth diagnostic investigation in the parents, which defined in one patient the paternal origin of the duplication, in one patient the de novo origin, and in one patient the maternal origin. The *SNTG2* gene encodes for a scaffolding protein that interacts with neuroligins (*NL4X* and *NL4Y*) as a crucial binding factor at inhibitory synapses. Mutations in the neuroligins (*NL3* and *NL4X*) have been implicated in the development of autism and ID [60]. In particular, it was observed that the binding of neuroligins to *SNTG2*, at inhibitory synapses can be influenced by mutations in neuroligins, showing how interactions between neuroligins and *SNTG2* could be implicated in the aetiology of autism [61]. Indeed, the SFARI database reports the *SNTG2* gene with a score of 2.1, Strong Candidate, indicating an unambiguous implication of the gene in ASD. Further screening studies in autistic individuals for the *SNTG2* gene could elucidate the interaction between *SNTG2* and neuroligins and ultimately the association with neurodevelopmental disorders.

Finally, another interesting gene that we have analysed and explored is the cholinergic receptor nicotinic alpha 7 (*CHRNA7*) gene, which is altered in six patients with ASD, ID and psychomotor developmental delay, two of whom have a parental origin. Our index case is an 11-year-old child with ASD, a speech delay with the inability to formulate complete sentences and express himself clearly, and a behavioural disorder characterized by immaturity in social interactions. We were able to detect a 459.63 kb duplication of maternal origin in region 15q13.3, which affects intron 3 to intron 9 and exon 4 to exon 10 of the *CHRNA7* gene. The *CHRNA7* gene encodes for the α7 subunit of nicotinic acetylcholine receptors (nAChRs). The nAChRs are highly expressed in the human brain and play a role in synaptic plasticity, learning and memory through calcium signalling at the neuronal level [62]. In particular, the *CHRNA7* gene is listed as a strong candidate in the SFARI database with a score of 2.1, making it an important candidate gene for neurodevelopmental disorders. Indeed, deletions and duplications of the *CHRNA7* gene have been reported in patients with neurological phenotypes such as cognitive deficits, epilepsy, language disorders, abnormal behaviour, ASD, ADHD or attention deficit disorder, mood disorders, and schizophrenia, with varying degrees of penetrance and severity, suggesting that the human brain is sensitive to *CHRNA7* dosage [63]. It could be hypothesized that altered *CHRNA7* dosage leads to changes in α7 protein abundance, which in turn alters calcium signalling in the corresponding areas of the central nervous system [64]. Studies conducted on iPSCs and human neural progenitor cells (NPCs) have shown that heterozygous deletion of the 15q13.3 region results in lower levels of *CHRNA7* reaching the cell membrane, leading to a decrease in channel activity, while duplication leads to an increased expression of *CHRNA7* with the accumulation of the misfolded protein and decreased trafficking of *CHRNA7* to the membrane with impaired calcium flux [65].

## 5. Conclusions

In recent years, great progress has been made in elucidating the aetiology of NDDs. The considerable percentage of familial cases associated with a high concordance rate in monozygotic twins sustains a genetic basis of these disorders [66]. On the other hand, the functional significance of CNVs affecting individual genes and/or genomic regions is still notably incomplete, making diagnostic processes and genetic counselling difficult [67]. Consequently, the discovery of significant genes that can be considered causative or associated with neurodevelopmental disorders represents a necessary step in the diagnostic procedure, for a better understanding the pathophysiology of NDDs [68,69]. A deeper knowledge will indeed help to better classify the clinical features of the patients and the mechanisms that underlie their clinical manifestations.

In this study, a-CGH allowed the detection of VOUS containing interesting genes potentially related to the clinical characteristics found in the patients analysed at our facility. In particular, we have been able to observe how the genes *CHRNA7*, *ANKS1B*, *ANKRD11*, *RBFOX1*, *ASTN2*, *GABRG3*, *SHANK2*, *KIF1A SETBP1*, *SNTG2*, *CTNNA2*, *TOP3B*, *CNTN4*, *CNTN5*, and *CNTN6* are involved in important phases of brain development and the correct functioning of the central nervous system. In particular, the presence of alterations in these genes in other patients with NDDs, availability of studies on animal models, and data from the literature make them interesting candidate genes for NDDs, essential to clarify and contribute to the knowledge of the molecular bases of these complex disorders.

For this reason, the results of our study, according to the literature, underline the significance and the usefulness of a-CGH in the genetic diagnosis. In fact, a-CGH offers significant sensitivity and resolution for the identification of submicroscopic chromosomal rearrangement and represents one of the most used techniques in daily practice [70]. However, since a-CGH still leaves some patients with NDDs undiagnosed, the interplay between a-CGH and NGS panels of known disease genes or exome sequencing (ES) can be considered an added value in aetiological diagnosis of these disorders [71,72,73,74]. Furthermore, in recent years, ES has been indicated in many studies as a first-line diagnostic test for NDDs, and for this reason, in the future, the integration of CNV calling algorithms into ES analysis could improve the characterization of complex phenotypes [75].

Considering the wide heterogeneity of NDDs, both in terms of behavioural and genetic features, further efforts are also needed to explore the possible role of environmental and epigenetic elements in phenotype modulation, and moreover, to better categorize each patient, future studies should necessarily be conducted in tight collaboration between neuropsychiatry divisions and genetics laboratories.

## Figures and Tables

**Table 1:** Pathogenic/Likely pathogenic CNVs associated with specific syndromes identified in patients with neurodevelopmental disorder. The number of patients analysed column indicates the number of patients in which the single alteration reported in the a-CGH results. The inheritance column reports the segregation/de novo nature of the single CNV reported in the result. ASD: autism spectrum disorder; NDD: neurodevelopmental disorder; ID: intellectual disability; ADHD: attention deficit hyperactivity disorder; ND: not determined.

Number of Patients Analysed	Reason for Referral	Array-CGH Results	CNV Classification	Inheritance	Related Syndrome/OMIM Number
7	ID, dysmorphic features, psychomotor development delay, ASD	arr 16p11.2 deletion	Pathogenic/Likely pathogenic	1 maternal origin 2 De novo 4 ND	16p11.2 Deletion syndrome (#611913)
9	ADHD, ID, ASD, speech delay	arr 16p11.2 duplication	Pathogenic/Likely pathogenic	3 De novo 6 ND	16p11.2 Duplication syndrome (#614671)
22	ID, psychomotor development delay, dysmorphic features, DiGeorge syndrome	arr 22q11.21 deletion	Pathogenic	1 paternal origin 6 De novo 15 ND	DiGeorge syndrome (#188400) Velocardiofacial syndrome (#192430)
15	Psychomotor development delay, learning deficit, growth delay	arr 22q11.21 duplication	Pathogenic	1 maternal origin 4 De novo 10 ND	22q11.2 microduplication syndrome (#608363) DiGeorge syndrome (#188400) Velocardiofacial syndrome (#192430)
8	ASD, psychomotor development delay	arr 15q11.2-q13.1 duplication	Pathogenic	2 De novo 6 ND	Duplication 15q11-q13 syndrome (#608636) Angelman syndrome (#105830) Prader–Willi syndrome (#176270)
10	Psychomotor development delay, ASD, ADHD, ID, psychomotor and growth delay, dysmorphic features, microcephaly	arr 1q21.1-q21.2 deletion	Pathogenic/Likely pathogenic	2 maternal origin 1 paternal origin 3 De novo 4 ND	1q21.1 Deletion syndrome (#612474) Thrombocytopenia-absent radius syndrome (#274000)
11	ASD, psychomotor development delay and epilepsy	arr 1q21.1-q21.2 duplication	Pathogenic/Likely pathogenic	1 paternal origin 4 De novo 6 ND	1q21.1 Duplication syndrome (#612475)
6	ASD, psychomotor development delay, Becker muscular dystrophy	arr Xp21.1 deletion	Pathogenic	6 ND	Duchenne muscular dystrophy (#310200) Becker muscular dystrophy (#300376)
5	ADHD, speech delay, Charcot–Marie-Tooth disease type 1	arr 17p12 duplication	Pathogenic	1 paternal origin 1 De novo 3 ND	Charcot–Marie-Tooth disease, type 1A (#118220)
4	NDD, psychomotor development delay, speech delay	arr 17p11.2 deletion	Pathogenic	1 paternal origin 3 ND	Smith–Magenis syndrome (#182290)

**Table 2:** Pathogenic/Likely pathogenic CNVs non-recurrent and not associated with syndromes identified in patients with neurodevelopmental disorder. The Number of Patients Analysed column indicates the number of patients in which the single alteration was reported in the a-CGH results. The Inheritance column reports the segregation/de novo nature of the single CNV reported in the result. ASD: autism spectrum disorder; ADHD: attention deficit hyperactivity disorder; ND: not determined.

Number of Patients Analysed	Reason for Referral	Array-CGH Results	CNV Classification	Inheritance
1	ASD	arr 11q13.1-q13.2 duplication	Pathogenic/Likely pathogenic	De novo
1	ASD	arr 5q14.3 duplication	Pathogenic/Likely pathogenic	ND
3	ASD, psychomotor development delay	arr Xq28 deletion	Pathogenic/Likely pathogenic	3 ND
1	ADHD	arr 13q21.32-q31.1 deletion	Pathogenic/Likely pathogenic	ND
9	ASD, psychomotor development delay, speech delay	arr 2p16.3 deletion	Pathogenic/Likely pathogenic	1 maternal origin 1 paternal origin 2 De novo 5 ND
1	Speech delay	arr 20p12.3-p12.1 duplication	Pathogenic/Likely pathogenic	ND
2	Psychomotor development delay, growth delay, ASD	arr 15q26.3 deletion	Pathogenic/Likely pathogenic	1 paternal origin 1 De novo

**Table 3:** The table show lists the most interesting variants of uncertain significance (VOUS) identified in our patients with neurodevelopmental disorders. The Number of Patients Analysed column indicates the number of patients in which the single alteration reported in the a-CGH results. The inheritance column reports the segregation/de novo nature of the single CNV reported in the result. These VOUS were selected on the basis of the score assigned to each gene in SFARI and the information present in the databases and literature. ASD: autism spectrum disorder; NDD: neurodevelopmental disorder; ID: intellectual disability; ADHD: attention deficit hyperactivity disorder; ND: not determined.

Number of Patients Analysed	Clinical Diagnosis	Array-CGH Results	CNV Classification	Inheritance	Gene OMIM Number and Score SFARI	Biological Function
12	ASD, psychomotor developmental delay, speech delay	arr 3p26.3-p26.2 deletion	VOUS	1 paternal origin 2 De novo 9 ND	*CNTN4* (*607280) Strong candidate	Essential for promoting dendrite growth and dendritic spine formation
1	ASD	arr 9q33.1 deletion	VOUS	De novo	*ASTN2* (*612856) Strong candidate	A central role in neural-glial adhesion during neuronal migration and synaptic strength through the trafficking and degradation of surface proteins
1	NDD	arr 18q12.3 duplication	VOUS	ND	*SETBP1* (*611060) High confidence	Role in controlling forebrain progenitor expansion and neurogenic differentiation
6	ASD, epilepsy, psychomotor developmental delay	arr 15q13.3 duplication	VOUS	1 maternal origin 1 paternal origin 4 ND	*CHRNA7* (*118511) Strong candidate	Involved in signal transmission at synapses
1	ASD	arr 11q13.3-q13.4 deletion	VOUS	ND	*SHANK2* (*603290) High confidence	Molecular scaffolds in the postsynaptic density of excitatory synapses
1	ASD	arr 2q37.3 duplication	VOUS	Paternal origin	*KIF1A* (*601255) Strong syndromic candidate	Microtubule-dependent motor protein responsible for fast anterograde transport of synaptic vesicle precursors in neurons
3	ASD	arr 15q12 deletion	VOUS	3 ND	*GABRG3* (*600233) Strong candidate	Major inhibitory neurotransmitter in the mammalian brain
3	ASD	arr 3p26.3 duplication	VOUS	1 De novo 2 ND	*CNTN6* (*607220) Strong candidate	Role in the formation of axon connections in the developing nervous system
1	ASD, epilepsy	arr 16q24.3 deletion	VOUS	ND	*ANKRD11* (*611192) High confidence syndromic	Regulates pyramidal neuron migration and dendritic differentiation in the developing mouse cerebral cortex
3	ASD, ID	arr 11q22.1 deletion	VOUS	1 maternal origin 2 ND	*CNTN5* (*607219) Strong candidate	Role in the formation of axon connections in the developing nervous system
5	ASD, ID, ADHD dysmorphic features	arr 12q23.1 deletion	VOUS	1 maternal origin 1 De novo 3 ND	*ANKS1B* (*607815) Strong syndromic candidate	Abundant in neuronal synapses and important role in normal brain development
8	ASD, ID, psychomotor developmental delay, speech delay	arr 2p25.3 duplication	VOUS	1 maternal origin 2 paternal origin 1 De novo 4 ND	*SNTG2* (*608715) Strong candidate	A scaffolding protein that interacts with neuroligins as crucial binding factor at inhibitory synapses
7	ASD, ID, psychomotor developmental delay, speech delay, dysmorphic features	arr 22q11.2 duplication	VOUS	1 paternal origin 6 ND	*TOP3B* (*603582) Strong candidate	Controls the DNA state during transcription by catalysing transient breakage and junction of a single strand of DNA
3	ASD, psychomotor developmental delay	arr 2p12 deletion	VOUS	1 maternal origin 1 paternal origin 1 ND	*CTNNA2* (*114025) Syndromic	Involved in the negative regulation of actin nucleation and in the regulation of neuron migration and the development of neuron projection
12	ASD, ID, ADHD	arr 16p13.3 deletion	VOUS	1 paternal origin 2 De novo 9 ND	*RBFOX1* (*605104) Strong candidate	Role in the regulation of the alternative splicing of large neuronal gene networks

## Data Availability

Data are contained within the text. Further details can be required to corresponding authors.

## Supplementary Materials

The following supporting information can be downloaded at: https://www.mdpi.com/article/10.3390/genes15040427/s1, Table S1: All CNVs observed in our study.
